# Diagnostic strategy for elderly patients with isolated greater trochanter fractures on plain radiographs

**DOI:** 10.1186/s12891-018-2193-5

**Published:** 2018-07-25

**Authors:** Nam Hoon Moon, Won Chul Shin, Min Uk Do, Seung Hun Woo, Seung Min Son, Kuen Tak Suh

**Affiliations:** 1 Department of Orthopedic Surgery, Pusan National University Hospital, Pusan National University School of Medicine, Busan, Republic of Korea; 2 Department of Orthopedic Surgery, Research Institute for Convergence of Biomedical Science and Technology, Pusan National University Yangsan Hospital, Pusan National University School of Medicine, 20 Geumo-ro, Mulgeum-eup, Yangsan, Gyeongsangnam-do 626-770 Republic of Korea

**Keywords:** Greater trochanter fracture, Occult intertrochanteric fracture, Diagnosis, Radiographs

## Abstract

**Background:**

Isolated greater trochanter (GT) fractures are relatively rare and few studies have assessed the appropriate diagnostic and therapeutic strategies for these fractures. When initial plain radiographs show an isolated GT fracture, underestimation of occult intertrochanteric extension may result in displacement of a previously non-displaced fracture. This study examined the clinical results and value of different diagnostic strategies in elderly patients with isolated GT fractures on plain radiographs.

**Methods:**

Between January 2010 and January 2015, 30 patients with initial plain radiographs showing isolated GT fractures were examined using MRI, bone scanning and/or CT for suspected occult intertrochanteric extension. We assessed the sensitivity, specificity, and positive and negative predictive value of each test. In addition, we noted the location of the fracture or soft-tissue injury on MRI in addition to treatment results.

**Results:**

All 30 patients had osteoporosis and fractures caused by minor trauma. MRI revealed isolated GT fractures in nine patients and occult intertrochanteric fractures in 21 patients. Using the MRI-based diagnosis as a reference, the results showed that plain radiographs, bone scans, and CT scans can be used for supplementary examination but they are not appropriate as confirmatory tests for these fractures. However, in patients with both isolated GT fractures seen on plain radiographs and increased uptake in only the GT area on bone scans, MRI revealed isolated GT fractures. The fractures were treated surgically in 20 patients and conservatively in 10 patients with satisfactory clinical results.

**Conclusions:**

We confirmed that MRI-based examination is useful in all symptomatic elderly patients whose plain radiographic findings reveal isolated GT fractures. However, we suggest that there is a need to establish a diagnostic strategy through increased understanding of the available diagnostic methods. We believe that surgical treatment should be considered in patients with occult intertrochanteric fractures that are detected on MRI.

## Background

Proximal femoral or “hip” fractures are a serious medical problem in orthopedic practice due to the increasing elderly population [1]. The approximate incidence of proximal femoral fractures is > 28,000 per year in the Korean population [2] and growing rapidly. Correct early diagnosis is very important for appropriate treatment and recovery in elderly patients because of a high first-year mortality rate (14–36%) and poor outcomes [3–5]. In 2–10% of patients presenting with symptomatic hip fractures, initial plain radiographs do not show the fracture line and are thus termed “occult fractures” [4]. Many epidemiological studies have reported that proximal femoral fractures are commonly related to osteoporosis that develops with age [2, 6]. Although isolated greater trochanter (GT) fractures are relatively rare [7, 8], the intertrochanteric extension of fractures has been demonstrated when plain radiographs show only an isolated GT fracture [8–10]. The diagnosis of occult intertrochanteric fractures of the femur on plain radiographs can be challenging, especially in elderly individuals with osteoporosis because of their poor bone quality [10, 11].

In most clinical institutes, the diagnosis of proximal femoral fractures is based on clinical history and physical examination, whereas confirmation is achieved by initial plain radiographs. However, when the initial plain radiograph shows an isolated GT fracture, underestimating the extent of the fractures may result in displacement of a previously non-displaced fracture, which will alter the treatment plan and necessitate more complicated surgery [3, 12]. Therefore, when an occult intertrochanteric fracture is clinically suspected, especially in elderly patients with osteoporosis, additional diagnostic examinations are necessary.

According to some recent reports, magnetic resonance imaging (MRI), which is becoming increasingly accepted and available, can more accurately define the anatomical extent of occult fractures and is routinely recommended for further evaluation in these situations [12, 13]. In addition, MRI is useful for the detection of soft-tissue injuries, especially in patients with clinical suspicion of occult intertrochanteric fractures [14]. However, the routine use of MRI for the detection of occult fractures is costly and requires a trained radiologist to review the images. Bone scans generally play a supplementary role in the detection of occult fractures but may produce false positive or negative results [15]. In addition, bone scans cannot show the precise extent of the fracture, often necessitating further imaging. Computed tomography (CT) scans are another alternative imaging option, and are more readily available and less expensive to perform. However, CT can lead to the misdiagnosis of occult hip fractures due to the resolution of the trabecular structure of severe osteoporotic bone compared with MRI [4, 16]. CT may also underestimate the full extent of injury in identified occult hip fractures [17, 18].

No protocol has yet been developed for the second-line investigation of isolated GT fractures identified on plain radiographs in elderly patients. The present study was designed retrospectively to examine the diagnostic strategy and clinical results in elderly patients with isolated GT fractures identified on plain radiographs. Therefore, we proposed the following questions: (1) what are the sensitivity, specificity, and positive and negative predictive value of each test and (2) what are the locations of the fracture or soft-tissue injury on MRI and results of treatment in these fractures.

## Methods

Between January 2010 and January 2015, we treated 455 consecutive patients with proximal femoral fractures affecting the femoral head, neck, GT, intertrochanteric, or subtrochanteric areas at our university hospital. Thirty patients with initial plain radiographs (anteroposterior, lateral, and Lorenz view) showing isolated GT fractures were examined by bone scans, CT, and MRI for suspicion of occult intertrochanteric extension. Patients with a definite intertrochanteric extension of the fracture line on plain radiographs were excluded. The patients were 65–91 years old (average 77 years) and 17 women (Table 1). Bone mineral density was measured in all cases preoperatively at an unaffected trochanteric region using dual-energy X-ray absorptiometry. Patient information was reviewed by the university human subjects committee, and Institutional Review Board approval was obtained prior to commencing the study.

**Table 1:** Demographic data of the patients

Number	Sex	Type of trauma	BMD (T-score)	Presence of pain	Presence of ecchymosis	Time from trauma to admission (days)
1	F	Slip in bathroom	−2.5	+	–	< 1
2	M	Slip in hospital	− 3.4	+	–	2
3	F	Slip in bathroom	−2.7	+	+	10
4	F	Slip in bathroom	−2.5	+	–	< 1
5	F	Slip in home	−4.0	+	–	< 1
6	F	Slip in home	−3.5	+	–	3
7	F	Slip in home	−3.9	+	–	< 1
8	F	Slip in home	−3.1	+	–	1
9	F	Slip in bathroom	−4.2	+	–	5
10	F	Slip in home	−2.8	+	–	< 1
11	F	Slip in home	− 2.6	+	+	< 1
12	M	Slip in hospital	−2.9	+	–	2
13	F	Slip in hospital	−2.5	+	–	13
14	F	Slip in home	−2.8	+	+	2
15	M	Slip in home	−4.0	+	–	7
16	F	Slip in home	−2.7	+	–	1
17	M	Slip in home	−2.5	+	+	0
18	F	Slip in home	−4.0	+	+	15
19	M	Bicycle accident	−3.0	+	–	16
20	M	Bicycle accident	−3.2	+	–	0
21	F	Slip in home	−3.1	+	–	4
22	M	Slip in home	−3.2	+	–	1
23	M	Slip in home	−3.5	+	–	1
24	M	Slip in home	−3.2	+	–	1
25	F	Slip in home	−3.5	+	–	19
26	M	Bicycle accident	−3.5	+	–	1
27	M	Slip in home	−3.5	+	–	2
28	F	Slip in hospital	−3.3	+	–	2
29	M	Pedestrian accident	−3.5	+	+	2
30	M	Slip in home	−3.5	+	–	< 1

*BMD* bone mineral density, *M* male, *F* female

**Fig. 1:**
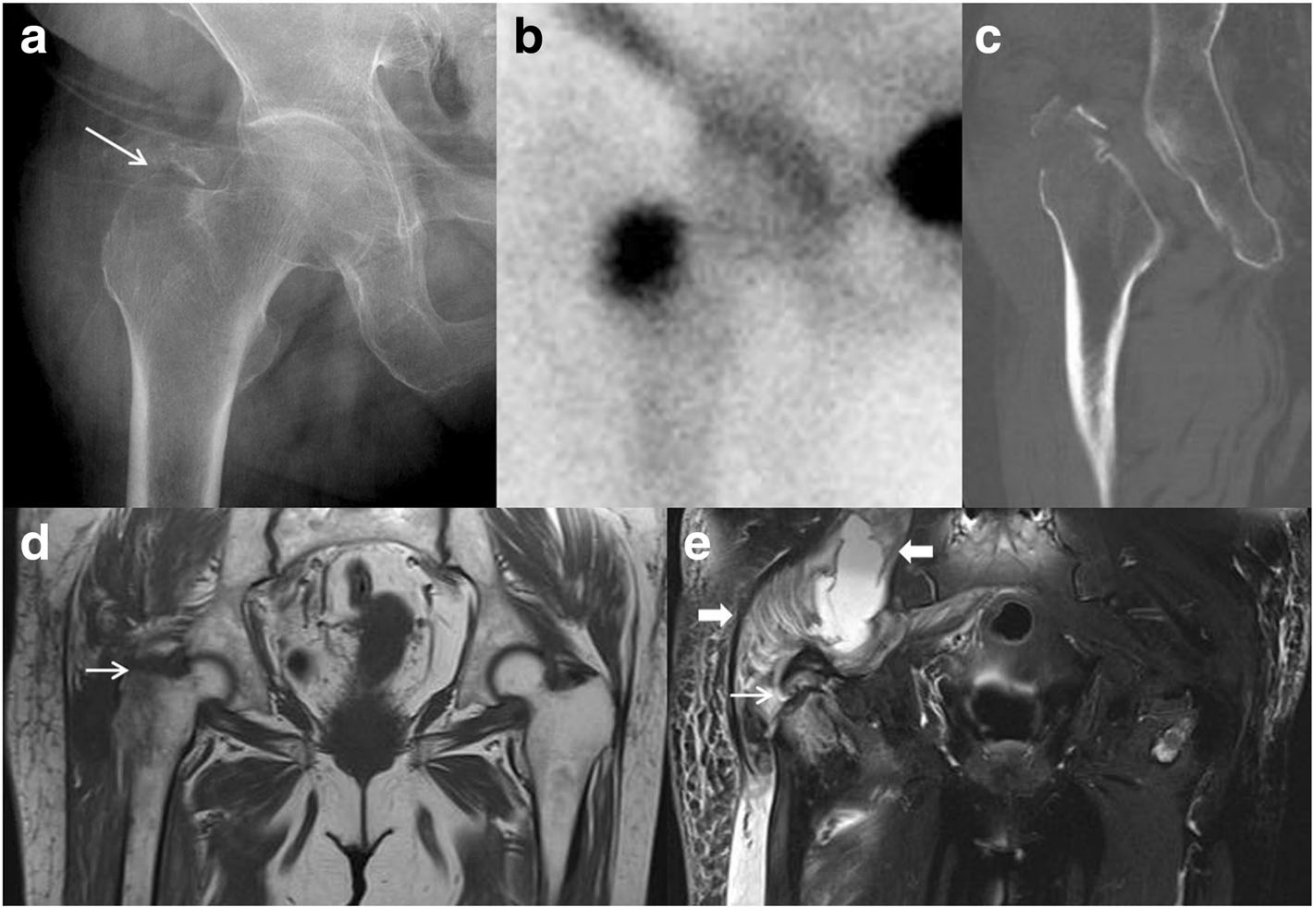
Case of a 72-year-old who presented at the outpatient department with right hip pain after slipping at home. **a** A plain radiograph of the right hip shows only an isolated greater trochanter fracture (*arrow*). **b** A bone scan obtained 3 days after the trauma shows radionuclide predominantly concentrated in the greater trochanter. **c** A computed tomography scan shows no occult intertrochanteric fracture. **d** A T1-weighted coronal image shows a linear low-signal intensity focus (*arrow*) indicating a fracture. **e** A T2-weighted coronal image shows a fracture surrounded by high signal intensity (*arrow*) and abnormal signal in the muscles around the hip. These findings are compatible with interstitial hemorrhage and hematoma (*bold arrows*)

MRI was performed at a mean of 5.3 days (range, 1–21 days) after trauma and was conducted on a 3.0-T magnet (MAGNETOM Verio, Siemens Healthineers) device using a commercially available pelvic phase array surface coil as a receiver. Sequence protocols included T1-weighted spin-echo in the coronal plane (slice thickness 3 mm with a gap of 0.5–1 mm, matrix 384 × 269) and axial plane (slice thickness 4 mm with a gap of 0.5–1 mm, matrix 384 × 176), and T2-weighted Turbo Inversion Recovery Magnitude in the coronal plane (slice thickness 3 mm with a gap of 0.5–1 mm, matrix 320 × 224), axial plane (slice thickness 4 mm with a gap of 0.5–1 mm, matrix 320 × 224), and sagittal plane (slice thickness 4 mm with a gap 0.5–1 mm, matrix 320 × 146). All MRI images were evaluated for the presence and extension of bone or soft-tissue injury. We determined the presence of a fracture when linear low signal intensity focus was surrounded by an intermediate signal on T1-weighted images and surrounded by high signal on T2-weighted images [19]. T2-weighted images facilitated the identification of soft tissue injuries and bone marrow edema [14, 20] (Fig. 1). The bone scan protocol consisted of both whole-body and hip pin-hole images. The bone scan was performed at a mean of 5.5 days (range, 2–21 days) after trauma. CT scans were performed using multidetector CT scanners (SOMATOM Definition Flash, Siemens Healthineers) with 128 detector rows. CT scanning from 2 cm cranial to the acetabulum and including the lesser trochanter distally was performed at a 2.0-mm slice thickness, and reconstructive images of the bone, soft, coronal, and sagittal views were obtained with high-spatial-resolution bone algorithms. CT scans were not performed in four patients because MRI was performed at the time of admission to the emergency room without additional CT scans. All MRI, CT, bone scans and plain radiographic images were reviewed by three observers (two authors and an experienced musculoskeletal radiologist).

We noted the location of the fracture or soft-tissue injury on MRI. Clinical instability was defined when a fracture line extended through more than 50% of the longitudinal axis on coronal images, and surgical treatment was conducted [10].

All data were collected in a structured database using SPSS software (version 20.0; IBM Corp.) Using MRI findings as the reference, the overall accuracy, sensitivity, specificity, and positive and negative predictive values (PPV, NPV) of each test were calculated. Categorical data were statistically analyzed by Fisher’s exact test. We performed receiver operating characteristic analysis to analyze and compare diagnostic performance between each test for occult intertrochanteric extension. In all tests, *P*-values < 0.05 were considered statistically significant. Data were reported with 95% confidence intervals.

## Results

Twenty-six of 30 (87%) fractures were caused by minor trauma that occurred indoors, such as through slipping. Only eight patients were admitted within 24 h after trauma; seven patients were admitted 7 days after trauma. All patients were elderly, > 65 years old, and had osteoporosis preoperatively (average T-score: − 3.2, range − 2.5 to − 4.2) (Table 1). Clinically, all patients presented with hip or proximal thigh pain. However, ecchymosis was evident in only six (20%) patients.

Initial plain radiographs of all 30 patients were interpreted as depicting minimally or non-displaced GT fracture. The types of fractures were finally identified by MRI (Table 2). Isolated GT fractures were present in only nine patients (30%) and occult intertrochanteric fractures in 21 patients (70%). In our overall data, the incidence of isolated GT fracture and occult intertrochanteric fracture was 2.0 and 4.6%, respectively, among 455 hip fractures in the same period. Seven patients’ bone scans showed increased uptake only in the GT region, 19 patients had extension into the intertrochanteric region, and four patients had normal findings. In 26 of 30 patients (87%), the MRI and bone scan results agreed. However, none of the bone scans definitively showed fracture propagation. On CT, 13 of 26 patients had isolated GT fractures, two had normal findings, and 11 had an occult intertrochanteric extension. In 20 of 26 patients (77%), the MRI and CT scan results agreed. Only 7 of 30 patients (23%) had isolated GT fractures on all imaging modalities (plain radiograph, bone scan, CT scan, and MRI) (Fig. 1).

**Table 2:** Radiologic findings and managements in the study group

Number	Plain radiograph	Uptake site on bone scan	CT	MRI	Presence of soft tissue injury	Type of management
1	Isolated GT fx.	No uptake	No data	IT fx.	+	Surgical
2	Isolated GT fx.	IT	No fx.	IT fx.	+	Surgical
3	Isolated GT fx.	No uptake	No data	IT fx.	+	Surgical
4	Isolated GT fx.	IT	No data	IT fx.	+	Surgical
5	Isolated GT fx.	IT	No data	IT fx.	+	Surgical
6	Isolated GT fx.	Isolated GT	Isolated GT fx.	Isolated GT fx.	+	Conservative
7	Isolated GT fx.	IT	No fx.	IT fx.	+	Surgical
8	Isolated GT fx.	IT	IT fx.	IT fx.	+	Surgical
9	Isolated GT fx.	IT	IT fx.	IT fx.	+	Surgical
10	Isolated GT fx.	IT	IT fx.	IT fx.	+	Surgical
11	Isolated GT fx.	IT	IT fx.	IT fx.	+	Surgical
12	Isolated GT fx.	IT	IT fx.	IT fx.	+	Surgical
13	Isolated GT fx.	Isolated GT	Isolated GT fx.	Isolated GT fx.	+	Conservative
14	Isolated GT fx.	IT	IT fx.	IT fx.	+	Surgical
15	Isolated GT fx.	IT	IT fx.	IT fx.	+	Surgical
16	Isolated GT fx.	IT	IT fx.	IT fx.	+	Surgical
17	Isolated GT fx.	No uptake	Isolated GT fx.	Isolated GT fx.	+	Conservative
18	Isolated GT fx.	IT	Isolated GT fx.	IT fx.	+	Conservative
19	Isolated GT fx.	Isolated GT	Isolated GT fx.	Isolated GT fx.	+	Conservative
20	Isolated GT fx.	IT	IT fx.	IT fx.	+	Surgical
21	Isolated GT fx.	IT	Isolated GT fx.	IT fx.	+	Surgical
22	Isolated GT fx.	IT	IT fx.	IT fx.	+	Surgical
23	Isolated GT fx.	IT	IT fx.	IT fx.	+	Surgical
24	Isolated GT fx.	Isolated GT	Isolated GT fx.	Isolated GT fx.	+	Conservative
25	Isolated GT fx.	No uptake	Isolated GT fx.	Isolated GT fx.	+	Conservative
26	Isolated GT fx.	Isolated GT	Isolated GT fx.	Isolated GT fx.	+	Conservative
27	Isolated GT fx.	IT	Isolated GT fx.	IT fx.	+	Surgical
28	Isolated GT fx.	Isolated GT	Isolated GT fx.	Isolated GT fx.	+	Conservative
29	Isolated GT fx.	IT	Isolated GT fx.	IT fx.	+	Surgical
30	Isolated GT fx.	Isolated GT	Isolated GT fx.	Isolated GT fx.	+	Conservative

*GT* greater trochanteric, *Fx.* Fracture, *IT* intertrochanteric

The diagnostic characteristics of the imaging modalities are shown in Table 3. Bone scans demonstrated the highest nominal sensitivity but low specificity for occult intertrochanteric fractures. The sensitivity of plain radiographs and CT scans was below 80%. The specificity was 77.8–100% and did not show significant differences across the three modalities (*p* = 0.157). The PPV was acceptable (≥80%) for most modalities, as was NPV for bone scans. In contrast, NPV was 69.2% for CT scans and only 30% for plain radiographs. MRI was the most accurate methodology, followed by bone scans, CT scans, and plain radiographs for occult intertrochanteric fractures. However, in patients with isolated GT fractures seen on both plain radiographs and bone scans, MRI also revealed isolated GT fractures.

**Table 3:** Diagnostic characteristics of the imaging modalities of occult intertrochanteric fracture of the femur (reported with 95% confidence interval)

	Plain radiograph (*n* = 30)	Bone scan (*n* = 30)	CT (*n* = 26)	*P*-value
Sensitivity	0.0 (0.0–22.8)	100.0 (77.2–100.0)	76.5 (50.1–93.2)	0.045
Specificity	100.0 (55.5–100.0)	77.8 (40.0–97.2)	100.0 (55.5–100.0)	0.157
Accuracy	30.0 (14.7–49.4)	93.3 (77.9–99.2)	84.6 (65.1–95.6)	0.046
PPV	NaN	91.3 (72.0–98.9)	100.0 (66.1–100.0)	
NPV	30.0 (14.7–49.4)	100.0 (47.3–100.0)	69.2 (38.6–90.9)	

*PPV* positive predictive value, *NPV* negative predictive value

**Fig. 2:**
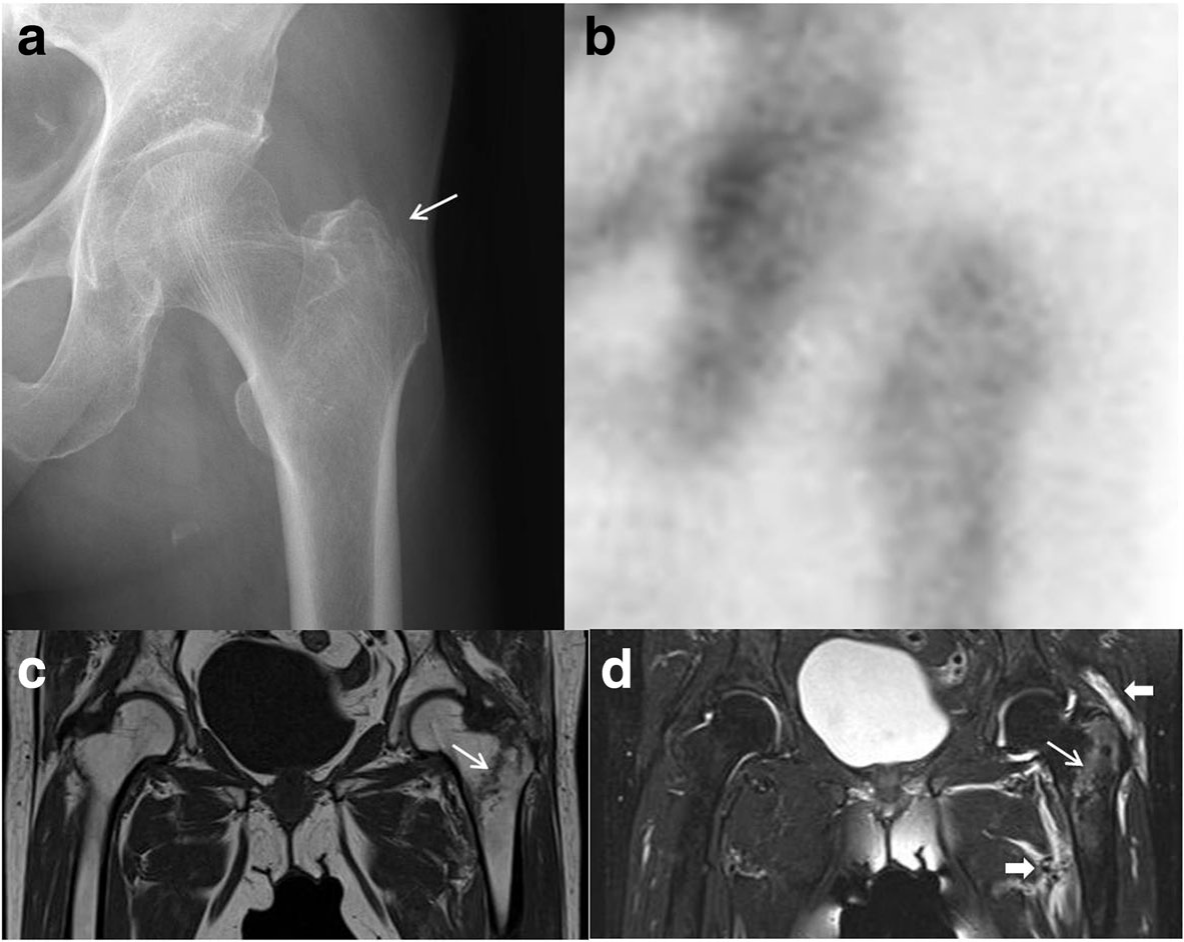
Case of an 84-year-old woman who presented at the emergency department with left hip pain after slipping in the bathroom. **a** A plain radiograph of the left hip only shows an isolated greater trochanter fracture (*arrow*). **b** A bone scan obtained 2 days after the trauma was unremarkable. **c** The T1-weighted coronal image shows an intertrochanteric fracture (*arrow*) that extends to the medial cortex. **d** The T2-weighted coronal image shows a fracture at the same level (*arrow*) and abnormal signal in the muscles around the hip (*bold arrows*)

**Fig. 3:**
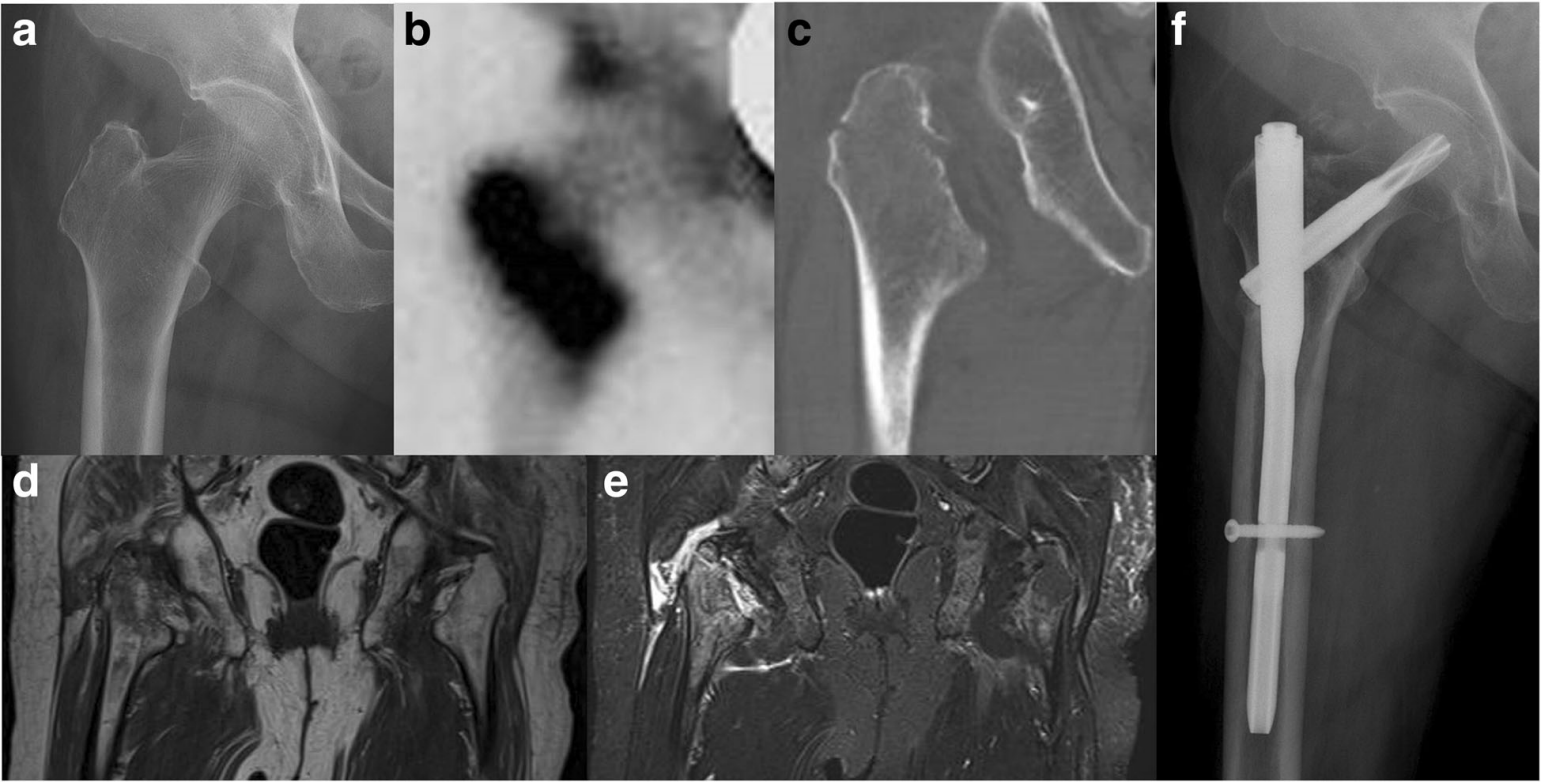
Case of a 73-year-old woman presented at the emergency department with right hip pain after slipping in a convalescent hospital. **a** A plain radiograph of the right hip shows an isolated greater trochanter fracture. **b** A bone scan performed 6 days after the trauma reveals that the fracture extended into the intertrochanteric region. **c** A computed tomography scan shows an isolated greater trochanteric fracture without intertrochanteric extent. **d, e** Magnetic resonance imaging shows an intertrochanteric fracture that crosses the midline to the medial cortex. **f** Internal fixation was performed using proximal femoral nail antirotation

On MRI images, soft tissue injuries such as hematoma or muscle injury were visible in all 30 cases (100%) with fracture (Fig. 2). Muscle edema, hemorrhage, and partial tears represented accounted for most muscle injuries of the obturator externus and the other external rotators, which include the gluteus maximus, piriformis, obturator internus, gemelli, and quadratus femoris. Nine patients with isolated GT fractures were treated conservatively and had satisfactory results, achieving bone union without any complications on follow-up radiographs. These patients commenced gradual weight bearing while ambulating with crutches or a walker after pain relief. Surgical treatment was conducted in 20 of 21 patients with occult intertrochanteric fractures using a proximal femoral nail (Fig. 3). All operations were performed by a single surgeon in the same technique using a Proximal Femoral Nail Antirotation (Synthes, Solothrun, Switzerland) system with the patient lying supine on a fracture table. No complications related to implant fixation were observed in the follow-up period. One patient was treated conservatively because she had high risks for surgical and anesthesia-related complications because of terminal lung cancer. All patients were instructed to walk with partial weight-bearing with the aid of crutches or a walker on the second day, with full weight-bearing as tolerated. Follow-up was possible in all 30 patients. The mean follow-up was 2.9 years (range, 1–5.5 years). The clinical results were good, as subjectively rated by both the patients and clinician. In this study, the first-year mortality rate of occult intertrochanteric fracture in elderly patients was 0% (Table 4).

**Table 4:** Clinical results in the study group

	Preoperative	Recovery
Charnley hip pain score^a^		4.9
Walking ability		
Independent community ambulator	16	14
Community ambulator with cane	8	9
Community ambulator with walker	2	1
Independent household ambulator	2	3
Household ambulator with cane	1	1
Household ambulator with walker	0	1
Nonfunctional ambulator	1	1
Death within 1 year		0

^a^Best possible score = 6 and worst possible score = 1

## Discussion

In the current study, we reviewed 30 patients with isolated GT fractures initially evaluated by plain radiographs and then further evaluated by either bone scanning, CT, or MRI. This retrospective, observational study describes 6 years of our experience with these fractures in elderly patients. We presented an efficient diagnostic strategy using the second-line investigations and integrated these data with a selection of established treatment methods.

The incidence of proximal femoral or “hip” fractures increases with age. Such fractures are associated with increased mortality [1, 3–5, 21]. Elderly patients are generally fragile and can experience hip fractures without major trauma. In addition, osteoporosis is strongly associated with increased risks for hip fracture [22]. Therefore, identifying osteoporosis as a potential risk factor for mortality after hip fracture is important because this can help to identify patients at high risk for mortality. Furthermore, hip fractures in elderly people with osteoporosis may present with occult or equivocal features due to poor bone quality [3, 6, 22]. For these reasons, correct early diagnosis is very important for the appropriate treatment and recovery of elderly patients with osteoporosis.

The demographic results of this patient series were consistent with those in other published studies [10, 12, 16]. The incidences of occult intertrochanteric femur fracture and isolated GT fracture among hip fractures were 4.6 and 2.0%, respectively. Only 9 of 30 patients (30%) had true isolated GT fractures on MRI. All fractures occurred in elderly individuals and all patients had osteoporosis preoperatively. Most fractures were caused by minor trauma that occurred indoors, such as that resulting from slipping. From these results, it is reasonable to assume that GT fractures with occult intertrochanteric extension are commonly related to osteoporosis because of clinical features, including increased incidence with age and associated minor trauma.

Most proximal femoral fractures are diagnosed based on clinical examination and a routine plain radiograph in the emergency department. However, in cases of occult intertrochanteric fracture of the femur, initial plain radiographs may show only GT fractures, especially in patients with osteoporosis [6, 9, 10]. To avoid the misdiagnosis of these fractures, second-line investigations such as bone scan, CT, or MRI are required. Bone scans have some advantages, including a short half-life, good availability, cost effectiveness, and relatively high detection rate (sensitivity 93%, specificity 95% [15]). As described by Matin [11], bone scans in elderly patients with closed fractures may appear normal if performed within 24 h of the injury. The interval from the injury to bone scan in this retrospective study was 2–21 days; therefore, our results have the advantage of excluding this problem. In patients with both isolated GT fractures seen on plain radiograph and increased uptake only in the GT area on a bone scan, MRI also revealed isolated GT fractures. Although our sample size was too small to determine the efficacy of bone scans, the technique is deemed to have diagnostic value due to the lack of false positive results in the present study. However, bone scans usually play only a supplementary role in the detection of occult fractures because they cannot show the precise extent of the fracture [12, 15]. In addition, bone scans may produce false negative results. In this study, four bone scans showed normal findings that represented false negatives. CT scanning is another alternative imaging option. Although CT excludes or verifies a fracture in most cases with inconclusive radiographs, CT is more likely than MRI to lead to the misdiagnosis of occult hip fractures. The evidence supporting the use of newly developed CT scanners with much better resolution than older scanners for the diagnosis of occult fractures is limited [4, 23, 24]. Lubovsky et al. [4] reported that CT led to misdiagnosis in 66% of patients with occult hip fractures, and noted that using CT before MRI caused a delay in diagnosis. Cabarrus et al. [24] reported that occult fractures were detected by 3D-CT in only 34 of 64 (53%) hips. Our results show that CT led to misdiagnosis in 6 of 26 (23%) patients despite using multidetector CT scanners with 128 detector rows. Failure to promptly diagnose the exact extension of fractures can result in the displacement of a previously non-displaced fracture, which can complicate the clinical situation.

Although MRI, an increasingly accepted and available method, is more expensive than other diagnostic imaging tools and susceptible to over-interpretation, it is recommended for the further evaluation and confirmation of occult hip fractures [4, 12–14, 19, 20, 25]. MRI has been considered as the “gold standard” in diagnosing occult and suspected proximal femoral fractures, with a reported sensitivity of 100%. It provides anatomical information as well as the identification of soft-tissue injuries [4, 16, 26, 27]. Using MRI diagnosis as a reference in this study, neither plain radiographs, bone scans, nor CT scans could be used as a single confirmatory test for the detection of occult intertrochanteric extension in elderly patients with osteoporosis. Of the 35 patients with fractures on MRI studied by Oka and Monu [14], 24 (69%) had muscle injuries such as edema, hemorrhage, muscle tears, or hematoma. Unlike their report, all patients in our series with isolated GT or occult intertrochanteric femoral fractures had accompanying soft tissue injuries. However, ecchymosis was evident in only six (20%) patients.

There are no standard treatment guidelines for occult intertrochanteric fractures of the femur. It is undisputed that isolated GT fractures in elderly patients are most often treated conservatively when they occur in isolation. The nine patients with isolated GT fractures in this study were treated conservatively and the results were clinically and radiographically satisfactory. Omura et al. [9] reported that seven patients with occult intertrochanteric fractures were treated conservatively with 1–3 weeks of bed rest followed by progressive walker-assisted ambulation. In a similar study, five patients with normal plain radiographs who had occult intertrochanteric fractures on MRI were managed conservatively [28]. The authors recommended that these patients with occult intertrochanteric fractures should be considered for conservative treatment. In contrast, in another study involving 10 patients with more complex injuries than apparently isolated GT fractures, six underwent surgery because of extension into the intertrochanteric region [29]. Schultz et al. [10] emphasized that incomplete intertrochanteric fractures that crossed the midline in the MRI coronal plane were treated surgically. Surgery was also carried out for all patients with no medical contraindications, regardless of the fracture pattern [12]. The authors determined that surgery for these patients focused on their underlying medical conditions and patient consent. Twenty of 21 patients with no medical contraindications underwent surgery because MRI showed extension into the intertrochanteric region through > 50% of the longitudinal axis on coronal images. In general, weight-bearing ambulation can cause incomplete intertrochanteric fractures to progress to complete fractures [28], which may lead to more complicated surgery, longer hospitalization, and delayed rehabilitation [3]. For these reasons, the authors prefer surgical internal fixation if possible. In the group of patients with occult intertrochanteric extension that received conservative treatment, one had a poor medical condition and refused surgery. The clinical results were subjectively good in all cases, and the first-year mortality rate of occult intertrochanteric fracture with osteoporosis was 0%.

This study had some limitations that should be discussed. First, the main limitation of this study is that it was retrospective in design. Second, the study population of 30 cases was too small to allow the general incidence of occult intertrochanteric femoral fracture and definitive criteria for treatment options to be established. Third, all images should be reviewed by different blinded radiologists with different experiences, and repeated measures should be obtained, in order to strengthen the research design. The investigation was, however, performed by an experienced senior experienced radiologist and the evaluation was performed together with two orthopaedic surgeons, limiting bias and the risk of false negative results. Finally, there was no control group to compare results between surgical and conservative treatment in patients with occult intertrochanteric fractures. The identification of more specific criteria for performing surgery or conservative management will require a larger prospective, case-control series and further analysis of the influence of fracture features on treatment options. As in Western countries, the number of Korean osteoporosis patients with proximal femoral fractures is increasing rapidly as the population ages. As a result, this study is thought to represent a valuable contribution to the literature despite the limitations described above.

## Conclusion

This study confirmed that MRI examination is useful in all symptomatic elderly patients when plain film radiographs show isolated GT fractures because of the inability of the film to reveal the geographic extent of the lesion, leading to questions regarding safe treatment. However, we suggest that there is a need to establish a diagnostic strategy through understanding the respective test methods. We also believe that surgical treatment and early ambulation in elderly patients is possible following the early detection of occult intertrochanteric fractures using proper diagnostic approaches.

## Abbreviations

CT: Computed tomography
GT: Greater trochanter
MRI: Magnetic resonance imaging
NPV: Negative predictive value
PPV: Positive predictive value
